# Ecosystem health and malfunctions: an organisational perspective

**DOI:** 10.1007/s10539-023-09927-9

**Published:** 2023-09-14

**Authors:** Emiliano Sfara, Charbel N. El-Hani

**Affiliations:** National Institute in Science and Technology in Interdisciplinary and Transdisciplinary Studies in Ecology and Evolution (INCT IN-TREE), Institute of Biology, Federal University of Bahia, Salvador, Brazil

**Keywords:** Organisational approach, Ecosystem, Health, Malfunction, Normativity

## Abstract

A recent idea of “ecosystem health” was introduced in the 1970s and 1980s to draws attention to the fact that ecosystems can become ill because of a reduction of properties such as primary productivity, functions and diversity of interactions among system components. Starting from the 1990s, this idea has been deeply criticized by authors who argued that, insofar as ecosystems show many differences with respect to organismic features, these two kinds of systems cannot share a typical organismic property such as health. In recent years, an organisational approach in philosophy of biology and ecology argued that both organisms and ecosystems may share a fundamental characteristic despite their differences, namely, organisational closure. Based on this kind of closure, scholars have also discussed health and malfunctional states in organisms. In this paper, we examine the possibility of expanding such an organisational approach to health and malfunctions to the ecological domain. Firstly, we will see that a malfunction is related to a lower effectiveness in the functional behaviour of some biotic components with respect to other systemic components. We will then show how some introduced species do not satisfactorily interact in an organisational closure with other ecosystem components, thus posing a threat to the self-maintenance of the ecosystem in which they are found. Accordingly, we will argue that an ecosystem can be said to be healthy when it is a vital environment organisationally grounded on its intrinsic capacity to ensure, under favourable conditions, appropriate functional behaviours for ecosystem components and ecosystem self-maintenance.

## Introduction

The notion of “ecosystem health”, albeit having older roots, began to gain popularity in the late 1970s and 1980s thanks to authors such as D. J. Rapport (e.g., Rapport et al. 1979, 1985), R. A. Ryder and C. J. Edwards (Ryder and Edwards 1985), and has been increasingly debated over the last years. According to Yang et al. (2019, pp. 5–13), publications on ecosystem health have shown a strong increase since 1989, and especially after 2006, with more than 350 publications on the subject appearing in 2018. Consequently, it is not surprising that this notion has also become the main topic of journals specifically devoted to it, such as *EcoHealth*,^1^*Ecosystem Health and Sustainability*,^2^*Aquatic Ecosystem Health & Management*,^3^*Journal of Aquatic Ecosystem Health*^4^ and *Ecosystem Health*,^5^ among others, as well as companies and associations that promote its dissemination, such as *EcoHealth Consulting* (EHC)^6^ and the *International Society for Ecosystem Health* (ISEH)^7^ (Rapport et al. 1999) and the *Aquatic Ecosystem Health and Management Society* (AEHMS).^8^

However, despite its influence, the notion of ecosystem health has also been much discussed (e.g., Ryder 1990; Costanza 1992; Suter II 1993; Rapport 1995; Lackey 2001; McShane 2004; Hearnshaw et al. 2005; Carolan 2006; Jax 2010), with a focus on a long-standing and still open issue, which is fundamental to the very notion, namely, whether it is really possible to attribute to ecosystems a typical organismic property such as health. In other words, one wonders whether an ecosystem can be indeed healthy or unhealthy.

It is well known that the term “health” is commonly used in everyday life to indicate a general state of well-being in a certain living entity, as well as to designate a correct “functioning” of a specific part of an organism. That is why one usually refers to a “healthy person”, “a healthy heart”, “a healthy stomach”, “a healthy liver” and so on. Evidently, this term has origins in the medical field (Conti 2018), where, in order to state that a person or organ is in “good health”, one tends to observe (or refer to) the internal functioning and/or the external symptomatology of a reference organism.

Apart from its terminological and medical links with organisms, a number of scholars believe that it is legitimate to extend the health concept to ecosystems as well. According to Yang et al. (2019, p. 6), David J. Rapport (e.g., 1989, 1992, 1995, and Rapport et al. 1979; Rapport et al. 1998; Rapport et al. 1999) and Robert Costanza (e.g., Costanza 1992; Costanza and Mageau 1999; Jørgensen et al. 2016) are probably the most representative scholars among those who contributed to introduce and develop a definition of “ecosystem health”. Rapport (1995, p. 293), in particular, argues that the ecosystem health concept “draws attention to the fact that ecosystems can become ill” as a result of a reduction of properties such as primary productivity, ecological functions, and the diversity of “interactions between system components” (Rapport et al. 1998, p. 397). He also suggests that the “methods of analysis and diagnosis developed in the health sciences can be appropriate in whole ecosystem contexts” (Rapport 1995, p. 293).

However, many doubts and criticisms have been raised regarding the notion of ecosystem health (e.g., Calow 1992; Suter II 1993; Wicklum and Davies 1995; Simberloff 1998; Lancaster 2000; Lackey 2001; Duarte et al. 2015). Lancaster (2000, p. 214), for instance, explicitly stated that the concept of health applied to ecosystems is a “a nebulous concept that should be expunged from the vocabulary”. The idea that an ecosystem can be sick or healthy would in fact presuppose, according to this author, that the ecosystem is endowed with biological features equivalent or almost equivalent to those of a living organism. For Lancaster (2000, p. 213) this would be not the case, which is why he does not hesitate to deem the idea of ecosystem health a “ridiculous notion”. For the same reasons, Simberloff (1998, p. 254) considers that “determining, at least to the satisfaction of all parties, whether an ecosystem is healthy is hopeless”. Along these lines, Calow (1992) argues that, since organisms and ecosystems have different biological characteristics, the concept of health would be hardly applicable to ecosystems. A similar criticism came from a well-known paper by Glenn W. Suter II, *A Critique of Ecosystem Health Concepts and Indexes* (1993). According to Suter II (1993, p. 1533), “ecosystems are not living organisms”, since “they do not behave like organisms and do not have properties of organisms such as health”. For this author, this means that health at the ecosystem level is mostly a metaphor, that is, a mere heuristic tool that human beings create for themselves in order to represent and understand ecological systems. Accordingly, there would be no objective basis to apply the concept of health to ecosystems. This notion would merely result from an analogical reasoning that improperly equates ecosystems and organisms in terms of their physical and biological characteristics. “The use of the phrase *ecosystem health*”, Suter II wrote, “is an attempt to draw metaphorically on the success and power of health science” (Suter II 1993, p. 1533).

With the present paper we aim to disprove this thesis. We will do this by firstly trying to point out, on the basis of recent studies appealing to an organisational approach (henceforth, OA), that despite their differences organisms and ecosystems may share a non-metaphorical, but objective characteristic, namely, organisational closure (Montévil and Mossio 2015). Second, based on this shared characteristic, we will attempt to show that health identifies an ecosystem organisational state that is far from being simply metaphorical, insofar as it is intrinsic to the very nature of both organisms and ecosystems.

For these purposes, we will trace the general theoretical lines of the organisational closure notion. As we will see in more detail in “General characteristics of the organisational approach” and “An organisational approach to ecosystems” sections, this expression identifies a mutual interaction between two or more parts, e.g., organs such as heart and stomach, which carry out a specific function serving the maintenance of both the parts and the whole system to which they belong, i.e., the organism. A stomach could not perform any digestive function without the cardiac function of a heart, which, in turn, in order to pump blood, cannot do without the digestive function of the stomach. In their circular relationship, the heart enables the functioning of the stomach and at the same time depends on it, and vice versa. Thus, the heart and stomach are related in such a manner that they are enabling and dependent on each other. The OA calls “organisational closure” this kind of circular relationship among enabling and dependent organismic parts. We will also see that the concept of organisational closure has already been used with respect to ecosystems, as a basis for ascribing functions to parts of an ecological system that are described as “constraints” and can be either biotic or abiotic items (Nunes-Neto et al. 2014; El-Hani and Nunes-Neto 2020). In general, constraints are parts of a system that, within a certain timescale, harness functional processes in a specific way that does not occur outside biological or ecological systems. Also, within a certain timescale, the properties that are relevant to their causal role within the system are not affected or altered by the processes they are harnessing.

As we will see in “Organism health, malfunctions and adaptive regulation” section, the health concept has been discussed in several works by authors associated with the OA, such as Saborido, Moreno and a number of colleagues (see Saborido et al. 2016). From an organisational point of view, we can state that an organism can be said to be in good health when one of the following two conditions occurs: (1) its constraints efficiently carry out the functions associated with its self-maintenance in a given environment, of which the organism is part. This means, also, that the organism has no “malfunctions” (Saborido et al. 2016) or, at least, no relevant malfunctions (i.e., malfunctions threatening the self-maintenance of the organism). (2) One or more malfunctions (both relevant and non-relevant) are compensated for by *adaptive regulation* mechanisms, which can thwart possible threats to the self-maintenance of the organism in question. These mechanisms are explained by the fact that the organism is characterised by an intrinsic *normative capacity*, which allows it to vary its internal physiological conditions according to the external context.

The original contribution of our study will be set out in the “Malfunctions and normative capacity in ecology: healthy ecosystems” and “Unhealthy ecosystems” sections, where we will apply the health concept originally developed by Saborido et al. (2016) to ecosystems.^9^ In our view, there are in fact examples of healthy ecosystems. This is the case when they appear to have no relevant malfunctions in their organisational structure (condition [1]). A key to looking at malfunction or lack of malfunction from an ecological perspective consists in considering the behaviour of a biotic component in an ecosystem. At the organismic level, the behaviour of a living being in an environment is (frequently) useful for its self-maintenance as an organism. At the ecological level, the same behaviour the organism performs for its self-maintenance may also play an ecological functional role, being useful for the ecosystem self-maintenance. Here is an example: at the organismic level, a bee’s behaviour of feeding on nectar contributes to its self-maintenance but at the same time also contributes to pollen transportation, making the bee also perform through its behaviour an important pollination function that plays a role in the ecosystem self-maintenance. If an organism is near death, unhealthy or sick to the point of not performing certain actions that are useful for its own sustenance, it may not thereby perform actions that are also ecologically useful. When this unhealthy condition affects a large number of organisms, this may jeopardise the ecosystem health as a whole, unless the ecosystem makes up for this deficiency with specific ecological adaptive mechanisms (condition [2]). When a decrease in one or more ecological functions threatens ecosystem self-maintenance, we can state that the ecosystem is not in a healthy state, and this claim is not simply metaphorical, as argued by critics of the ecosystem health notion: it has “naturalised” features (Moreno and Mossio 2015, p. 36) in the sense that it is an occurrence inherent to the ecosystem itself. It is therefore in “naturalised” terms (Moreno and Mossio 2015, p. 36), not merely metaphorical ones, that in the following pages we will try to understand the ecosystem health concept.^10^

## General characteristics of the organisational approach

By the expression *organisational approach*, we specifically refer to an approach in the philosophy of biology that has given rise to a number of theoretical developments from the late 1990s onwards (e.g., Schlosser 1998; Collier 2000; Bickhard 2000, 2004; McLaughlin 2001; Christensen and Bickhard 2002; Delancey 2006; Edin 2008; Mossio et al. 2009; Barandiaran et al. 2009; Ruiz-Mirazo and Moreno 2012; Moreno and Mossio 2015; Bich 2016; Saborido et al. 2016; Frick et al. 2019).^11^ In order to define the theoretical-biological conditions for a correct application of the organisational theory to organisms, Frick et al. (2019, p. 103) claimed that “the OA characterizes living systems as organised in such a way that they are capable to self-produce and self-maintain while in constant interaction with the environment”. Likewise, according to Moreno and Mossio (2015, p. 71), “organisation” occurs when a set of organic parts interact in a coordinated way in order to collectively support one or more “functions”. Such functions are conceived as intrinsically rooted in biological systems, not only in changing viewpoints of an external observer. In this regard, Moreno and Mossio (2015, p. 36) state that they are “naturalised”. These functions contribute to the global self-maintenance of an organism conceived as a whole that autonomously interacting with a given milieu.

Let us give a short example of function from an organisational perspective, using the classical case of the heart function. The heart has the function of pumping blood insofar as this pumping ability contributes to the maintenance of a living organism by making blood circulate and, thus, distribute gases and nutrients through the body, while contributing to the excretion of metabolic wastes, enabling in this manner the activity of any other organismic function, such as, say, the stomach digestive function, as well as other functions exerted by the liver, lungs, brain, and so on. At the same time, an organism’s heart is produced and maintained by every single functional part of the organism considered as a whole, that is, through the contributions of functions exerted by the brain, liver, lungs, stomach, and so forth. From an organisational perspective, the typical heart “whump-whump” sound is not a function, since, unlike the blood-pumping function, the heart “whump-whump” sound does not play any role, in the proper sense, in the *maintenance of* the organism: even if there was no such sound, or a different sound, every living being would maintain itself alive. From this perspective, following Mossio et al. (2009) and Moreno and Mossio (2015), the heart blood-pumping action is a function to the extent that (1) it contributes to the organism self-maintenance even at the time scale in which the heart does not pump blood (insofar as the heart pumps blood only at the instant when the heartbeat occurs), and (2) to the extent that it is also maintained by the contributions of other functions exerted by other organismic parts. For instance, if in a given organism we consider the heart and the stomach, on the one hand, the heart is part of the cause of the existence and functions of the stomach, while, on the other hand, without an organ dealing with food digestion, that is, without a stomach, the heart would not be able to perform its blood-pumping function. The same could be said if we consider, instead of the stomach, an organ like the brain. In this case, the brain is part of the cause of the existence and functions of the heart, giving rise to a circular relationship also between these two parts. This specific kind of relationship is what the OA calls “organisational closure” (Montévil and Mossio 2015). In general terms, a biological organism is, as a whole, an “organisationally-closed” and “thermodynamically-open” system (Moreno and Mossio 2015, p. 6). It is characterized by an organisational closure involving all its functional parts. These parts support a number of functions contributing to maintaining the whole system, namely, the organism itself, as well as all its components.

It is important to note that, according to the organisational theory, the functional parts of an organisationally-closed system are “constraints” (see, e.g., Moreno and Mossio 2015; Montévil and Mossio 2015). Constraints are parts of a system that, within a certain timescale, harness functional processes in a specific way that does not occur outside biological or ecological systems. Also, within a certain timescale, the properties that are relevant to their causal role within the system are not affected or altered by the processes they are harnessing. For instance, in organisms, the way oxygen flows through the bloodstream due to the vascular system is different from the way oxygen would flow in the open air or even within the body in the absence or failure of the vasculature, and at the time scale in which this harnessing effect on the flow of oxygen takes place, the vasculature is not affected in the relevant properties to its causal role. The vasculature can then be said to act as a constraint at that specific time scale (Montévil and Mossio 2015, p. 182).

## An organisational approach to ecosystems

In recent years, the concept of organisational closure and the organisational approach to function have been applied to ecosystems as well (e.g., Nunes-Neto et al. 2014; El-Hani and Nunes-Neto 2020). Indeed, as in the case of organisms, the ecosystem constraints, which are self-generated within the ecosystem itself, interact in order to establish mutual dependence, that is, a certain organisational closure, which in turn can be called “closure of constraints” (El-Hani and Nunes-Neto 2020, p. 71; see Montévil and Mossio 2015). In a closure of constraints, a given constraint enables the production of at least one other constraint within the system and at the same time depends for its production on at least one other constraint. By being involved in a closure of constraints, each constraint—as both enabling and dependent—is both cause and effect of the existence and activity of other constraints, and, when the concept is applied to ecological systems, of the whole ecosystem (El-Hani and Nunes-Neto 2020). Ecosystem constraints include both biotic items (organisms, species, functional groups, etc.) and abiotic factors, such as fire or habitat heterogeneity, on the condition that the specific abiotic factor at stake is under the control of the ecological system, being both an enabling and dependent constraint, and thus included in its closure of constraints (El-Hani and Nunes-Neto 2020; El-Hani et al. forthcoming). This condition on functional ascription to abiotic factors is important for avoiding an excessive liberality in the attribution of ecological functions according to the organisational theory.^12^ By requiring that ecological function be ascribed only to constraints that are both enabling and dependent within a given system, the theory establishes clear criteria for proper and improper functional ascription to both biotic and abiotic factors (El-Hani et al. forthcoming).

Following El-Hani et al. (forthcoming), consider, in a given ecosystem, a key function of plants, which is to carry out (through photosynthesis) the fixation of carbon atoms initially contained in atmospheric carbon dioxide molecules in more complex compounds such as glucose. Once the flow of carbon atoms is harnessed or constrained in this manner, carbon becomes part of plant biomass, and, then, herbivorous feeding on that biomass realize a second channelling of the flow of carbon atoms, such that the carbon atoms found in plant biomolecules become, after digestion, absorption of nutrients, and synthetic pathways, part of herbivorous bodies. Then, when the life cycle of consumers (such as herbivores) and producers (such as plants) finishes, the animal carcasses, as well as plant leaves, fruits, and twigs become part of the organic matter that is further processed by the constraining action of decomposers (fungi, bacteria, etc.), which in turn convert this matter into available nutrients for plants, closing the circular relationship among constraints. In fact, such a circular closure involves a mutual dependence among the constraints (plants, herbivores and decomposers). By constraining the flow of carbon atoms, the producers (plants) create conditions of possibility to, i.e., enable the existence of the decomposers and, at the same time, exert a fundamental effect on the ecological system considered as a whole. On the one hand, producers are enabling conditions to the existence of decomposers. On the other hand, decomposers depend on the producers, since it is from the producers that they obtain part of the matter and energy necessary for their own self-maintenance. And the same goes to the other elements in the example. In summary, plants, herbivores and decomposers exert specific functions whose mutual dependence contribute to the self-maintenance of the whole ecosystem.

As mentioned above, ecological functions can also be ascribed, based on the organisational theory, to abiotic constraints. Let us take the case of fire. According to El-Hani et al. (forthcoming), fire can be considered a functional constraint when it is under the control of other constraints internal to the ecological system, namely, when it can be described as both enabling and dependent, being part of the ecosystem closure of constraints. On the contrary, when fire is not, at the same time, enabling and dependent, it will be an external boundary condition that contributes to the ecosystem’s dynamics, but is not part of its core organization. Fire can be enabling, on the one hand, when it favours or prevents the growth or reproduction of certain plant species in a manner that contributes to the maintenance of other constraints within the ecosystem as well as to ecosystem self-maintenance. On the other hand, it can be dependent on fire-adapted plant species. This is the case of some Eurasian ecosystems that are characterised by the presence of trees belonging to the *Pinaceae* (pine) family, such as larch (*Larix *spp*.*) and *Pinus sylvestris*. Archibald et al*.* (2018) indicate that these two species tend to effectively resist high-intensity crown fires: “larch is deciduous, and the two pine species shed their dead lower branches, so that when fire occurs it usually only spreads in the understory without reaching the canopy” (Archibald et al. 2018, p. 5). Black and Bliss (1980) claim that this kind of surface fires in such Eurasian ecosystems probably kill juvenile spruces (*Picea spp*., whose structure and bio-composition favour, by contrast, intense crown fires) before they reproduce, thus contributing “to maintain the species composition and fire regime of the region” (Archibald et al. 2018, p. 5). In this sense, vegetation is a real driver of fire regimes, which allows us to speak even of a coevolution of fire and biota (McLauchlan et al. 2020). When it is under the control of biotic constraints (like plants, which are organisationally interconnected with other biotic constraints such as herbivores and decomposers) internal to the ecological system, and when it is both enabling and dependent, an abiotic constraint such as fire contributes, as in the example just given, to the self-maintenance of the ecosystem in question.

## Organism health, malfunctions and adaptive regulation

The idea of organism health in the OA has been mainly developed by Saborido (2012), Moreno and Mossio (2015), Saborido and Moreno (2015), and Saborido et al. (2016). From an organisational viewpoint, we can say that a state of health occurs when a given organism does not present relevant malfunctions, or when the functions carried out by its internal constraints properly contribute to the maintenance of the organism conceived as a whole. By “relevant malfunction”, we mean a malfunction that threatens the self-maintenance of an organism. There is a difference between a relevant malfunction, like an acute peritonitis, and a mild malfunction, like an excessive and persistent production of sebum causing slight symptoms of seborrheic dermatitis in some humans. The latter case, unlike the former, is not necessarily a sign of poor health, as we will explain in detail below, since it does not threaten the organism’s self-maintenance. This usually implies that the organism does not experience hindrance in its daily activities of interacting with an environment, in particular, those activities that are fundamental to its self-maintenance. For example, it does not experience any strong sensation of muscle pain while in locomotion.

It is important to outline, vice versa, that in the presence of relevant malfunctions an organism finds it more difficult to self-maintain itself, that is, to carry out a number of vital activities in its external milieu that play a central role in its self-maintenance. In this case, the organism, as already argued by the philosopher of medicine Georges Canguilhem, is not in a healthy state, since its “normative capacity” (Canguilhem 1991, p. 183) to concretely interact with the environment is reduced. To put it otherwise, the organism faces difficulty in performing (or is no longer able to carry out) actions that, within a given environmental (or socio-environmental) context,^13^ it was previously able to perform in order to self-maintain without significant difficulties.

This last point is stressed by Saborido et al. (2016), who state that biological organisations naturally instantiate normativity, which is inherent to their very nature as such, to the extent that normativity is intrinsic and deeply rooted into biological functioning. As they write, “life is in fact a normative activity” (Saborido et al. 2016, p. 116). In Saborido and colleagues’ work, normativity is conceived as the living organism’s capacity to autonomously respond to the “changing demands of the environment” (Saborido et al. 2016, p. 116), effectively adapting itself to the external context variability. Accordingly, a healthy state should not be simply assessed by the organism’s aptitude to be statistically “normal” with respect to a statistical or populational average (as stated, e.g., by Boorse 1977): health should be assessed by the specific and individual normative capacity through which organisms effectively adapt to their vital context (Canguilhem 1991, p. 181).^14^ Indeed,

the organisational interpretation of “correct functional behaviour” is very different from the concept of “normal function” […] We [do not] need to appeal to a “reference class” or to an “idealized type” […] to justify when an organism is functioning incorrectly. The normativity of organisational malfunctions is based on the organisational properties of each token living being. (Saborido et al. 2016, p. 115)

The Canguilhemian sense of “normativity” adopted by Saborido and colleagues deviates from how this term is usually used in the literature on function and health. Although it does not exclude at all the common idea (or “dominant view”, as pointed out by Wouters 2005, p. 124) of normative function as something that a specific item is “supposed” to do (for instance, a heart is supposed to have the function of pumping blood, see, e.g., Saborido et al. 2016, p. 102), in this sense “health” more specifically indicates the capacity, typical of living organisms, to properly vary their internal physiological organisation during interaction with the external context. The average heart rate of a human being, for example, increases when the organism goes from a state of rest to a state of more intense and constant movement such as running. The heart increases its beats in response to the increased demand of oxygen by the muscles. This causes a subsequent reorganisation of other internal physiological traits, such as increased respiratory rate and increased sweating (Hawley et al. 2014). Similarly, when faced with an unexpected dangerous situation, the heartbeat accelerates, blood vessels in muscles dilate, digestion in the gastrointestinal tract is inhibited, pupils dilate, release of glucose from the liver increases. (Powley 2003; see also Saborido et al. 2016, p. 108). These are adaptive mechanisms allowing to effectively interact with the context when the organism’s behaviour in that context changes (such as when we start running) or when the socio-environmental scenarios themselves change (such as when a dangerous situation suddenly arises).

Along these lines, the organisational point of view assesses malfunction by considering, at the same time, the functional organisation of a given living being’s internal parts, and the success of that same living being in adapting effectively to the outside world. To put it otherwise, saying that an organism presents one or more malfunctions entails considering the specific milieu in which that organism lives. An ordinary pneumonia could be a clear example of malfunction, insofar as pneumonia generally identifies an inflammatory condition of the lung representing an evident obstacle to the vital activity of an organism, in particular with regard to its self-maintenance. Likewise, although not as deadly as an ordinary case of pneumonia, a chronic gastritis frequently leads the individual to have a diet not as varied as in the earlier stages of his or her life. Simply put, as a result of the emergence of malfunctions, the organism’s ability to adapt to the environment is lower than before, and the same can be said, accordingly, of its ability to self-maintain itself in the set of environments with which it interacts. It is in this particular sense that we can state that such an organism has a “lower” normative capacity: it displays a reduced ability to adapt to a given milieu or certain circumstances, and thus a reduced ability to self-maintain. An individual close to death from a fatal disease or a deep wound near the heart, in all likelihood, has an almost zeroed normative capacity.

As characterized by normativity, biological organisms are endowed with mechanisms of “adaptive regulation” (Saborido et al. 2016, p. 106; Di Paolo 2005, p. 438). It is because of this sort of phenomena that the bio-physiological conditions within the living being can vary when a certain context changes, or as a result of a particular concrete activity in a certain context, in order to preserve the organism’s self-maintenance. For example, the homeostatic acceleration of a human being’s heartbeat during a certain sporting activity represents an adaptive regulation mechanism.^15^

Often, adaptive regulation mechanisms, precisely because they aim to maintain the integrity of the biological system, can compensate for malfunctions. Saborido et al. (2016, p. 114) indicate some case studies in which such mechanisms can be detected. One of these cases is a heart condition called *mitral valve prolapse* (MVP) (see Delling and Vasan 2014). Generally speaking, mitral valve prolapse is the swelling of one or both mitral valve flaps in the left atrium during heart contraction. But “a person with a malfunctioning prolapsed mitral valve […] can remain asymptomatic and with excellent cardiac function throughout all of his or her life thanks to myocardial adaptation mechanisms” (Saborido et al. 2016, p. 114). What occurs in asymptomatic cases of MVP is an adaptive change at the level of the left ventricular myocardium, resulting in a morpho-functional alteration of the cardiac valve and the extent of the prolapse. Indeed, as indicated by Sutton and Sharpe (2000, p. 2981), an adaptive remodelling of the left ventricular myocardium can be detected in case of valvular heart disease. This adaptive change enables the organism to maintain good cardiac performance, which in turn enables it to be self-maintained as a whole. Such a condition, when it does not lead to a significant reduction of the subject’s capacity to concretely act in a certain external context (i.e., when the subject normally carries out the daily activities he/she used to do before this heart condition onset), does not represent a case of malfunction from an organisational perspective (Saborido et al. 2016, pp. 114–115).

Similarly, we are dealing with an adaptive regulation phenomenon when a biological system shifts to a different regime of organisation in which a potentially harmful event, or a complete or partial interruption of a function,^16^ can integrate with other internal traits according to this new regime of organisation. For instance, the complete or partial interruption of the function of light transduction in the eyes triggers a new organisational state (insofar as, for instance, a sharpening of other sense organs such as hearing can occur—see Petrus et al. 2014) that may not affect global self-maintenance (Saborido et al. 2016, p. 107). Simply put, a living being with irreversible blindness or low vision can certainly stay alive, being capable of self-maintenance by successfully coping with its environment. But, according to the organisational point of view we are adopting, both blindness (involving a complete interruption of visual function) and a myopic state (involving a partial interruption of visual function) represent cases of malfunction, since the general normative capacity of the organism is reduced compared to the vital state prior to the pathological state onset. Nevertheless, what is at stake here is that such pathological manifestations involve the triggering of a series of adaptive mechanisms avoiding the self-maintenance breakdown. In this case, insofar as the organism can maintain itself as a whole over a certain period of time by concretely interacting with its external environment without further difficulty, and despite a malfunctional phenomenon in the visual system, the organism is in good health. Therefore, it can be claimed that malfunction, when counteracted by adaptive regulation mechanisms, does not necessarily imply disease or illness at the level of entire organism.^17^

In the case above, MVP is not a malfunction because it does not significantly damage cardiac function, and, thus, the entire organism can be said to be not ill but in health, insofar as it continues to self-maintain and to efficiently interact with its external environment. Differently, despite the adaptive mechanisms at stake, the states of blindness or myopia involves a clear reduction in the effectiveness of the visual function, for which they can both be considered malfunctions of the visual system. But, yet, thanks to adaptive mechanisms or to a new organisation regime, the organism in question continues to self-maintain and to efficiently interact with its external environment as a whole, thus keeping itself in a state of health.

Finally, we underline that there are physiological manifestations in which the disorder of a function is not detected as harmful by a specific organism, triggering no adaptive regulation mechanism. In such instances, in the absence of other relevant pathological conditions within the biological system, it can be said that the organism is healthy, since its physiological-organisational state does not show any significant malfunction signs. As discussed by Saborido et al. (2016, p. 113), Gilbert’s syndrome provides a medical example of a morphological and functional alteration that does not generate a compensatory adaptive reaction. Gilbert’s syndrome is a benign liver disorder in which the liver does not properly process bilirubin, a pigment contained in the bile (Wagner et al. 2018). About one-third of patients affected by this condition are nearly asymptomatic. From an organisational point of view, this is due to the absence of malfunctioning occurrences linked to an above-average concentration of bilirubin in the blood: both organisational structure and self-maintenance of the organism are not affected by this kind of alteration during the interaction with the context in which it lives. Since the organism does not experience hindrance in its daily activities, not experiencing relevant pain or malaise sensations, and to the extent that it has no relevant malfunctions at its physiological (and organisational) level, it can be said to be healthy.^18^

## Malfunctions and normative capacity in ecology: healthy ecosystems

In order to apply the organisational notions of health and malfunctions to ecosystems, let us now consider—as a case in point—the specific functions that may result from the activity or behaviour of the so-called introduced species within a given ecological context.

Introduced species, often referred to as “alien species”, “exotic species”, “foreign species”, etc.,^19^ are more or less large groups of organisms living in ecosystems other than their native habitat, as a direct or indirect consequence of human activities (Richardson et al. 2000). It is well known that a vast amount of species once “alien” is now an integral part of many Earth ecosystems (Thompson 2014) without generating any damage to the environment, despite the fact that it has been believed otherwise for a long time (Sagoff 2005). As Davis et al. (2011, pp. 153–54) wrote, after the “devil’s claw” plant (*Martynia annua*: see Hevly 1969), native to Mexico and introduced to Australia in the nineteenth century, was for years steadily eradicated “probably as a horticultural oddity”, local people began to let it grow wild, with the result that now “it does not substantially change the fundamental character of its environment by […] reducing biodiversity or altering nutrient cycling”. Besides, human beings have always used a certain amount of alien species parts (e.g., non-native trees’ wood) for purely practical purposes or for food. Kiwifruit, for instance, is considered a delicacy by many people around the world. Kiwifruit, also known as “kiwi”, is the edible berry of plants of the *Actinidia* genus, which constitute an example of introduced species insofar as they are widespread elsewhere than in their native region: China. Some *Actinidia* species (in particular, *Actinidia deliciosa*) located in New Zealand have been the subject of a relatively recent study (Pomeroy and Fisher 2002) showing that, within specific local ecosystems, the kiwifruit production is largely due to the pollination activity of honey bees, mainly *Apis mellifera*, and bumble bees, mainly *Bombus terrestris*. Both *A. mellifera* and *B. terrestris* are in turn introduced species, since, although now widespread in many parts of the world, the former is probably native to Africa (Whitfield et al. 2006) or Asia (Han et al. 2012), while the latter is probably native to Europe, North Africa, and the Near East (Rasmont et al. 2008).

Pomeroy and Fisher (2002**)** state that the pollination process by *B. terrestris* is particularly effective for kiwifruit production. This kind of process, which we should call more properly pollination function, allows the reproduction of New Zealand *Actinidia*, whose nectar is the food helping to keep alive the same insects from which it benefits. This is an ecological case of closure of constraints, in which *B. terrestris* and *Actinidia* are the constraints, while pollination and nutrition are the related functions. *B. terrestris* is enabling since the function it performs (pollination, in the present case) contributes to the reproduction process and thus to the self-maintenance of *Actinidia*. At the same time, *B. terrestris* is dependent on *Actinidia*, since the latter nourishes *B. terrestris*, thus contributing to its self-maintenance, and vice versa. Insofar as in this specific kind of organisational closure a mutual self-maintenance between two constraints is observed, the ecological closure in question shows no malfunction. As we broaden our analysis to include ever larger or more complex levels of closure, up to ecosystems, most of the literature we consulted (e.g., Macfarlane and Gurr 1995; Goulson and Hanley 2004; Newstrom-Lloyd 2013) indicate that the presence of *B. terrestris* in various New Zealand ecosystems seems to contribute to the conservation of a number of biodiversity items, insofar as *B. terrestris* is an important pollinator not only of kiwifruit, but also of passion fruit, feijoa, and cucurbits (Macfarlane and Gurr 1995, p. 33). In fact, *B. terrestris* pollinates spring-flowering fruit, berry, and nut crops during adverse weather conditions, when other bee species are mostly inactive, and “it also pollinates at least 53 species of native plants from coastal to subalpine areas” in New Zealand (Macfarlane and Gurr 1995, p. 33).

On the basis of the latter cases, it can be said that *B. terrestris*, insofar as it ensures more than sufficient pollination and plant reproduction, contributes to the self-maintenance of several New Zealand ecosystems. Therefore, in this case it is an introduced species, but, as it does not harm the native ecosystems and their biodiversity, it is not invasive. Moreover, the native ecosystems can be said to remain in a healthy state in its presence. When a New Zealand ecosystem can maintain itself in a relatively stable or resilient state^20^ over a certain period of time (showing an adequate capacity for self-maintenance), it shows no signs of malfunctioning from the organisational viewpoint we adopt. The introduced species, for its part, is not a cause of malfunction. This is explained in view of the fact that the normative capacity levels of organisms inhabiting the ecosystems prior to the introduction of the alien species are equally guaranteed: the alien species in this case does not harm native biodiversity. In these instances, the ecosystem is healthy, since it has no malfunctions in its organisational structure. It persists in a healthy state because the living beings within it remain in a healthy state, in such a manner that they keep their functional contributions to ecosystem self-maintenance.

The reason certain ecosystems are able to integrate introduced species such as *B. terrestris* could be due to two (perhaps concomitant) causes, which could in turn be the subject of further study in the future: (I) The ecological emergence of a set of adaptive regulatory mechanisms^21^ similar to adaptive regulation phenomena in organisms (we refer to our example above concerning MVP, “Organism health, malfunctions and adaptive regulation” section; (II) Alien species are not harmful for the ecosystem in question, as in the case above of Gilbert’s syndrome in human beings (“Organism health, malfunctions and adaptive regulation” section).

In the first case, the behaviour of some bumble bees that allows them to nibble at plant leaves in order to facilitate flower production if pollen is scarce (Pashalidou et al. 2020) could constitute an adaptive regulation case at the ecological level. This is a case where adaptive regulation occurs thanks to a specific behaviour of the bee (leaf nibbling), in which, in order to satisfy its own nutritional needs, the bee fosters the production of a certain amount of food (flower pollen) that would otherwise be lacking. As a food source, pollen contributes to the self-maintenance of the bee, which can continue to exercise its function as a pollinator in the ecosystem, which in turn continues to self-maintain. Similarly to the MVP example, in which adaptive regulation (left ventricular myocardium remodelling) prevents the onset of a possible cardiac malfunction caused by the anomalous swelling of one or both mitral valve flaps, this ecological example also shows a regulation that takes place at the level of the adaptive behaviour of an ecosystem constraint (the bumble bee), which continues to perform an effective pollination function despite the occurrence of certain unfavourable conditions (scarce pollen) that could have hindered this function by generating a malfunction.

In the second case, which coincides with the above-mentioned example of devil’s claw (Davis et al. 2011, p. 153), a new non-native species does not pose a threat to the self-maintenance of the ecosystem in which it is introduced. Similar to the case of Gilbert’s syndrome (in which the anomalous processing of bilirubin by the liver does not generate a compensatory adaptive reaction), the spread of devil’s claw in Australia, since it does not interfere with the functional processes allowing the self-maintenance of the ecosystems, does not give rise to adaptive regulation phenomena. An even more striking example of this second case is that of *B. terrestris* in New Zealand ecosystems. The ecological behaviour of B*. terrestris*, although exogenous, significantly contributes to the conservation of a number of biodiversity item due to an effective pollination function, which not only does not interfere but even increases the beneficial effects for the ecosystem when other bee species are mostly inactive (Macfarlane and Gurr 1995, p. 33). In contrast to case (I), in case (II) no specific mechanism of adaptive regulation is detected. But in both cases (I and II), the ecosystem continues to self-maintain without showing significant changes in its biodiversity, nutrient cycling, fundamental ecosystem functions, or else. It is in this sense that the ecosystem can be considered in health.

In addition, there could be a final case (III) that could be compared, once again, to the organismic case, in which a potentially harmful phenomenon can integrate with other internal traits according to a new regime of organisation (as in the example above of the complete or partial interruption of the eyes’ function of light transduction, “Organism health, malfunctions and adaptive regulation” section). Goulson (2003, p. 1) indicates that in “New Zealand many weeds from Europe are […] visited by European honeybees and bumblebees” and that “introduced bees are primary pollinators of a number of serious weeds”. Let us consider the hypothetical case of such a New Zealand ecosystem in which weeds increase. Weeds are known to have a negative impact on several plants (see, e.g., Sardrood and Goltapeh 2018). However, following our hypothetical case and considering the previously mentioned literature that does not consider *B. terrestris* as dangerous for New Zealand ecosystems, the vital processes of these plants might not show significant slowdowns in terms of growth, reproduction, and self-maintenance. This could be due, say, to defensive mechanisms of allelopathy, which consists in the release, by the plants, of biochemicals capable to cope with the harmful effect of weeds. For instance, as plants release phenolic acids, they reduce the activity of phenol-β-glucose transferase, thus inhibiting the root growth of weeds (Cheng and Cheng 2015). This allelopathy case is, according to the organisational theory we are adopting, an example of adaptive regulation, allowing us to assert that the ecosystem can effectively cope with a malfunction in the regular growth, reproduction, and self-maintenance of plants constituting its biotic community. In this third case, just as in case (I) and (II), the ecosystem can be said, again, to be healthy. As in case (I) and unlike case (II), in case (III) the emergence of adaptive regulation mechanisms is detected. However, unlike case (I), in case (III) these mechanisms do not prevent the presence of a malfunction. They rather allow the system to self-maintain *in spite* of a malfunction, just as it occurs in the case of a partial interruption of the eyes’ function of light transduction, which does not necessarily threaten the self-maintenance of an organism. In the ecological example under consideration, the release of plant biochemicals that weaken the weeds does not eliminate the malfunction, namely, the very presence of the weeds. However, due to the triggering of regulatory mechanisms that allow the constraints to effectively adapt to the harmful effect of the malfunction, the system is not threatened in its self-maintenance and is, therefore, healthy.

## Unhealthy ecosystems

Several studies indicate that, in geographical areas in China, Australia, South America, Canary Island, Israel, and South Africa, *B. terrestris* is strongly suspected of causing potential damage to ecosystems (see, e.g., Dafni and Shmida 1996; Kondo et al. 2009; Acosta 2015; Naeem et al. 2018). That is, in these ecosystems *B. terrestris* is not only an introduced but also an invasive species. This is due to its damaging interactions with local bee fauna, given its “competition for nest sites with, and genetic contamination of, local *Bombus* spp., spread of parasites and pathogens” (Dafni et al. 2010, p. 101). Some of these negative effects have been empirically shown. For example, Ne’eman and Dafni (1999), Ne’eman et al. (2000), Hingston et al. (2004) and Kenta et al. (2007) showed an actual reduction in seed production of local flora, with a decrease in pollination processes, due to impacts related to *B. terrestris*. Sometimes, this is due to the tendency of this species to steal different food sources from other bees or insects, which may be, then, geographically excluded (Acosta 2015). A smaller number of bees or insects implies a lower pollination activity, which can have ecosystem consequences. As indicated by Lundgren et al. (2013), although a decline in pollinator availability does not necessarily cause negative effects in some plants, other plants may be affected by this decline, for example, by showing fecundity decrease. In the latter cases, we are faced with both a loss of ecological pollination function and a marked reduction in the normative capacity levels of organisms living in the ecosystem, which may affect ecosystem self-maintenance, for instance, through effects such as reduction in biodiversity due to the local extinction of some species or reduction in plant fecundity. In this case, adaptive regulation mechanisms may not be available at the ecosystem or community level, or these mechanisms may be too weak to be effective. From an organisational point of view, in this case one can speak of ecosystem malfunction related to pollination processes. At the same time, precisely because of this significant reduction in the normative capacity levels within it, it can be said that the ecosystem is not in a healthy state.

An even more striking case of ecosystem malfunction linked to a reduction in organisms’ normativity is found in Redonda, a small volcanic Caribbean Island. In this case, the reduction resulted from a sharp decrease in biodiversity levels. This has been related to the introduction of black rats (*Rattus rattus*) and domestic goats (*Capra hircus*) in Redonda at some time in the late 1500s (Donihue et al. 2020). These introduced and invasive species had a “devastating effect on the island, resulting in the loss of nearly all trees and most of the ground vegetation” (Donihue et al. 2020, p. 379), and in the near total annihilation of at least four endemic lizard species, such as the ground lizard (*Pholidoscelis atratus*). Rats and goats damaged several island ecological functions through the direct deletion of much of the plant and animal biodiversity. Indeed, as shown by Coblentz (1978), the feeding behaviour of *C. hircus* often leads to degradation or annihilation of much of the vegetation on which it feeds. In parallel, as indicated by Donihue et al. (2020), the black rats that populated Redonda, by constantly feeding on lizards, drastically reduced their numbers. As the number of plants was drastically reduced, the pollination function was also damaged or compromised. It follows that the recursive relationship between a given insular plant and a pollinating insect (as constraints showing mutual relationships, within a closure of constraints) was itself damaged. A case of ecological malfunction ensued, therefore, in Redonda Island’s ecosystems (or even, we might say, in Redonda Island as a whole ecosystem). Human intervention, which had initially triggered this malfunction through the introduction of two non-native species, has been subsequently used to attempt to return the island to a state of newfound health, thanks to an ecological restoration programme implemented in 2017, which initiated a rapid regrowth of both native vegetation and invertebrate populations (Donihue et al. 2020).

It is worth emphasizing that, in the Redonda Island example, a drastic decrease in ecosystem biodiversity levels does not lead to a healthy new ecosystem, despite the presence of new species populating it, such as goats and rats. In fact, the biodiversity was depleted to such an extent that an adequate support of the ecological functions necessary for the self-maintenance of a new ecosystem could not be guaranteed. As shown by Donihue et al. (2020), insofar as it lacked both a large number of organisms that previously populated it and the related ecosystem functions they performed, the island ecosystem under consideration was no longer healthy, as the spread of goats and rats threatened its self-maintenance.

The organisational theory of health we propose can be applied to many ecosystem examples, related not only to cases of introduction of exotic species or to perturbations triggered by human activity. Eutrophication, a well-known process leading to a massive reduction of biodiversity in certain aquatic environments due to an excessive concentration of nutrients (such as nitrogen and phosphorous), is one such example. While eutrophication often results from pollution, it is not the case that it is always due to human action. A lake, for instance, may be “naturally eutrophied when situated in a fertile area with nutrient-enriched soils” (Nasir Khan and Mohammad 2014, p. 2), or sometimes due to the fact that lake environments themselves may “naturally lose their self-purification capacity over certain decades” (Akinnawo 2023). In a lake, certain ecological constraints such as the primary producers (plants, algae) play a feeding function in relation to other biotic components, such as consumers (e.g., fish). In turn, fish, as constraints acting in an organisational closure with plants, play an egestion function that contributes to several nutritional needs of the plants, as fish excrements are remineralised by bacteria, which make the nutrients present in faecal matter available to the primary producers as a food source (Villéger et al 2017, p. 791). As a result of eutrophication, the increase in nutrients leads to an overgrowth in the number of primary producers in the lake ecosystem, resulting in a multiplication of the aerobic bacteria that feed on them. Gradually, these bacteria cause a drastic decrease in oxygen levels, leading to the death of the fish. Again, the recursive relationship between primary producers, such as plants, algae and fish is disrupted, leading to the death of the latter and to a consequent threat to the self-maintenance of the ecosystem, which is therefore unhealthy.

Previously (“Organism health, malfunctions and adaptive regulation” section), we argued that the kind of normative capacity underlying the health concept is related to organisms considered as wholes, i.e., as agents interacting with the contexts where they live, not simply to organisms’ internal constraints. In ecosystems, organisms play a constraining role, as reciprocally linked and self-generating parts internal to such systems. We would like to stress now that, although what is at stake in this paper is the ecosystem health concept, the specific kind of normative capacity to which we refer does not change: it relates to organisms as wholes, namely to organisms as functional agents in an environmental milieu. Once organisms are characterized as constraints within ecosystems, based on the organisational theory of ecological functions, we need to look at the biotic parts found in a specific ecosystem, i.e., organisms and their way of acting and behaving in an environment, in order to refer to a normative capacity.

In the last examples, ecosystems invaded by *B. terrestris* or by goats and rats or ecosystems in eutrophied states does not appear to be healthy. It can be said that they are in a “pathological” state (Smith 1984; Rapport 1989). But as pointed out by the organisational approach (Saborido et al. 2016), what is pathological is not opposed to what is commonly defined as “normal”, but precisely to what is “normative”. Normativity can be assessed by the ability of living beings to live healthily within a given ecosystem, as well as by the ecosystem stability and resilience. Thus, health is an expression of normativity, that is, of the organisms’ capacity to interact effectively with the ecosystem environment, and, accordingly, to contribute to ecosystem self-maintenance. A heavily decreased biodiversity, or a persistent mortality among species (Donihue et al. 2020), is in our view a concrete index to assess the healthy or pathological state of an ecosystem.

## Concluding remarks

In general terms, organisation is nothing more than the property of systemic components interacting with each other in order to compose and regulate, within a certain period of time, the system of which they are part. Insofar as it is possible to speak of health when the organismic parts efficiently perform the functions maintaining the organism in a normative interaction with the environment (as argued by Saborido and colleagues, see “Organism health, malfunctions and adaptive regulation” section), in our view it is also possible to speak of health when the system in question is an ecosystem: an ecosystem is healthy when the biotic elements composing it are themselves sufficiently healthy such that they can satisfactorily carry out the functions allowing the ecosystem to maintain itself (say, pollination, dispersion, regeneration, and so forth), fulfilling effectively the natural norms underlying ecosystem self-maintenance. A conspicuous reduction in biodiversity levels, resulting from the local extinction of many biotic components in an ecosystem, may make the self-maintenance of the ecological system difficult or even impossible. Along these lines, the arguments presented in this paper support the conclusion that both healthy and pathological states can be attributed to ecosystems on sound theoretical grounds, based on the organisational theory, such that we can argue that, even if ecosystem health is regarded, as Suter II (1993) argues, as a metaphor, it will still be a fruitful metaphorical model, or, alternatively, that ecosystem health is not merely a metaphor, but a concept that can be consistently and naturalistically grounded on a theoretical account of ecological systems.

We can establish, thus, what we precisely mean when we state that ecosystem malfunction should be conceived in *naturalised* terms: (1) on the one hand, malfunction is intrinsically rooted in ecosystems that fail to achieve self-maintenance; (2) on the other hand, a given ecological component or function (such as *B. terrestris* or its pollination activity) may be malfunctioning in certain ecosystems, but not malfunctioning in others, since every ecosystem has particular characteristics (e.g., a particular community structure, species composition, etc.) that make it different from any other ecosystem.

In the present paper, we tried to deal with general concepts allowing to establish a basic analogy between organisms and ecosystems when one talks about health, based, in particular, on the notion of organisational closure. Indeed, we agree with Rapport in that, in order to speak of ecosystem health, “it is not essential […] that the systems being compared be identical in all respects. What is crucial is that they share some properties” (Rapport 1995, p. 304). Thus, against the above-mentioned critics of the notion of ecosystem health (e.g., Suter II 1993; Calow 1992; Simberloff 1998; Lancaster 2000), there is no need to assume that ecosystems are organisms to use concepts like health to account for ecosystem features. But, aside from analogies, it is obvious that there are numerous differences between organisation in ecosystems and organisms, and, also, between the ecological mechanisms of adaptive regulation mentioned above and organismic adaptive regulatory mechanisms, which will be worth investigating. For this reason, we are aware that in scientific practice there will be contexts in which one should distinguish between organisms’ and ecosystems’ specific characteristics, where the undeniable differences between organisms and ecosystems may imply a more restricted or shrewd use of the approach to the concept of ecosystem health we exposed. After all, our arguments above tended to lump organismic systems and ecological systems together with respect to their organisational traits. In certain scientific practices, it might be possibly appropriate to resort to studies that, while postulating an ecosystem health notion, tend to keep such a notion separate from any general concept of organismic health or not to make excessive use of this concept (e.g., Jørgensen et al. 2016; Costanza and Mageau 1999). Actually, we do not doubt the scientific and philosophical legitimacy of such approaches. Just like our approach, and each in their own way, such studies provide an idea of health fitting well with ecosystem functioning. Just like our approach, such studies provide theoretical tools to evaluate, in a general way, whether an ecosystem is healthy or not. The choice of our or other such approaches may be determined by the specificity of the working contexts in which scientists or philosophers of science carry out their inquiry practices as well as by the specific nature of the ecosystems they are investigating. While having used a theoretical slant, we therefore hope that our approach may come in handy for epistemic purposes (Ludwig 2016), i.e., for establishing to what extent such an organisational framework of ecosystem health may be fruitful in practical-scientific contexts related to ecology, biology, zoology, and so on. This is another point we aim to explore in the future.

## Footnotes

1 Website: https://www.springer.com/journal/10393.

2 Website: https://www.tandfonline.com/journals/tehs20.

3 Website: https://www.tandfonline.com/journals/uaem20.

4 This journal changed its name to *Journal of Aquatic Ecosystem Stress and Recovery* in 1997 and was incorporated into in *Hydrobiologia* 2002. Website: https://link.springer.com/journal/10813/.

5 The last volume of this journal was published in 2009. Website: https://onlinelibrary.wiley.com/journal/15260992.

6 Website: http://www.ecohealthconsulting.com/index.html.

7 This association was founded in 1994 and dissolved in 2006: https://uia.org/s/or/en/1100049153.

8 Website: https://aehms.org/.

9 For reasons of space, the health concept thus understood will not be connected, in this paper, with other relevant yet complex notions such as ecosystem robustness and resilience. Any similarities or differences among these concepts and their applications will remain to be explored in further studies. On some theoretical links between ecosystem health and resilience, see, e.g., Jax (2010) and Berkes et al. (2012).

10 It is possible that one prefers to use other terms than “health” to refer to states of ecosystems that are degraded or unbalanced (or not), such as “ecosystem viability” (e.g., Raphael and Marcot 1994) or “ecosystem disruption” (Montévil 2021). However, we think that the term “health” has an additional meaning that could be lost if we opted for these other terms, namely, “health” entails a normativity component related to organisms, allowing us to differentiate normal from pathological states in a stronger manner that has consequences in the domain of the environmental sciences.

11 As indicated by Moreno and Mossio (2015), the organisational approach has its theoretical roots in some classical authors such as Immanuel Kant, and also in some more recent thinkers such as Maturana and Varela (1980) and Piaget (1967), among several others.

12 It is worth noting that, in addition to the organisational theory, other philosophical approaches have been developed to account for ecological functions. On this point and, specifically, on the differences between the organisational approach and other accounts of ecological functions, see Nunes-Neto et al. (2014), Cooper et al. (2016) and Lean (2021).

13 A similar conception of health as a person’s ability to perform intentional actions and achieve goals has been formulated in the case of humans by Nordenfelt (2007, p. 6), who points out that such a conception goes back to Galen (second century AD).

14 As Canguilhem (1991, p. 183) puts it, “the animals whose thermoregulation has lost all its usual flexibility and who are incapable of struggling for their food or against their enemies are normal only in laboratory surroundings where they are sheltered from the brutal variations and sudden demands of adapting to the environment […]. The sick living being is normalized in well-defined conditions of existence and has lost its normative capacity, the capacity to establish other norms in other conditions”.

15 Bich et al. (2015) developed a concept of “regulation” that does not include homeostatic phenomena of cardiac regulation. The “adaptive regulation” concept referred to in the present article is more general and broader. We will deal elsewhere with Bich and colleagues’ notion of regulation and how it may or may not be applied to ecological systems.

16 In organisational terms, a complete interruption of a biological function in organisms can be also referred to as “dysfunction” (Moreno and Mossio 2015, p. 82).

17 In a strict sense, malfunction is not an attribute of the whole organism, i.e., we can only attribute a malfunction to the internal constituents of an organism. Notice that also in common language it is not appropriate to say that an organism is malfunctioning, but that it is sick or ill.

18 The manifestation of a certain illness may, however, not depend on malfunctioning episodes (Saborido et al. 2016). A major depressive disorder (Culpepper et al. 2015), for instance, is often due to social factors linked to the patient’s experiential life. It does not generate any malfunction in the sense described above, since it does not affect the organism’s morpho-functional state.

19 On the use of these and related terms, see Colautti and MacIsaac (2004).

20 We conceive the concepts of stability and resilience here in a very broad sense, i.e., as referring to the capacity of an ecosystem to absorb variations that could potentially change its fundamental characteristics and, thus, to remain in certain dynamic regimes. Certainly, these concepts deserve more analysis, but we should leave this task to be pursued elsewhere, due to space constraints and also because it would make us deviate from the goal of this paper.

21 See Sterelny (2005) for a philosophical discussion of compensation effects.
